# *Toxoplasma gondii *infection and liver disease: a case-control study in a Northern Mexican population

**DOI:** 10.1186/1756-3305-4-75

**Published:** 2011-05-13

**Authors:** Cosme Alvarado-Esquivel, José Luis Torres-Berumen, Sergio Estrada-Martínez, Oliver Liesenfeld, Miguel Francisco Mercado-Suarez

**Affiliations:** 1 Faculty of Medicine, Juárez University of Durango State. Avenida Universidad S/N. 34000 Durango, Dgo, Mexico; 2 Mexican Social Security Institute, Avenida Normal # 200, 34000, Durango City, Durango, Mexico; 3 Institute for Scientific Research, Juárez University of Durango State. Avenida Universidad S/N. 34000 Durango, Durango. Mexico; 4 Institute for Microbiology and Hygiene, Campus Benjamin Franklin, Charité Medical School, Hindenburgdamm 27. D-12203 Berlin, Germany; 5 Roche Molecular Diagnostics, Pleasanton, CA. USA

## Abstract

**Background:**

Infection with the protozoan parasite *Toxoplasma gondii *may cause liver disease. However, the impact of the infection in patients suffering from liver disease is unknown. Therefore, through a case-control study design, 75 adult liver disease patients attending a public hospital in Durango City, Mexico, and 150 controls from the general population of the same region matched by gender, age, and residence were examined with enzyme-linked immunoassays for the presence of anti-*Toxoplasma *IgG and anti-*Toxoplasma *IgM antibodies. Socio-demographic, clinical and behavioral characteristics from the study subjects were obtained.

**Results:**

Seroprevalence of anti-*Toxoplasma *IgG antibodies and IgG titers did not differ significantly in patients (10/75; 13.3%) and controls (16/150; 10.7%). Two (2.7%) patients and 5 (3.3%) controls had anti-*Toxoplasma *IgM antibodies (*P *= 0.57). Seropositivity to *Toxoplasma *did not show any association with the diagnosis of liver disease. In contrast, seropositivity to *Toxoplasma *in patients was associated with consumption of venison and quail meat. *Toxoplasma *seropositivity was more frequent in patients with reflex impairment (27.8%) than in patients without this impairment (8.8%) (*P *= 0.05). Multivariate analysis showed that *Toxoplasma *seropositivity in patients was associated with consumption of sheep meat (OR = 8.69; 95% CI: 1.02-73.71; *P *= 0.04) and rabbit meat (OR = 4.61; 95% CI: 1.06-19.98; *P *= 0.04).

**Conclusions:**

Seropositivity to *Toxoplasma *was comparable among liver disease patients and controls. Further studies with larger sample sizes are needed to elucidate the association of *Toxoplasma *with liver disease. Consumption of venison, and rabbit, sheep, and quail meats may warrant further investigation.

## Background

Human infection with the protozoan parasite *Toxoplasma gondii *occurs worldwide [1,2]. Major routes of *T. gondii *infections include ingesting food or water that is contaminated with oocysts shed by cats or by eating undercooked or raw meat containing tissue cysts [2-4]. The clinical spectrum of *T. gondii *infection varies from an asymptomatic state to severe illness. The parasite can affect the host's lymph nodes, eyes, central nervous system, liver, and heart [3,5,6]. In liver, the parasite has been associated with a number of pathological changes including hepatomegaly, granuloma, hepatitis, and necrosis [7-14]. In addition, an epidemiological study has reported an association of *T. gondii *infection with liver cirrhosis [15]. However, epidemiological studies on the association of infection with *T. gondii *and liver disease are scarce, and have not been performed in Mexico. Therefore, we performed a case-control study in Northern Mexico to determine the seroprevalence of *T. gondii *infection and anti-*T. gondii *IgG levels in adult patients with liver disease attending the Department of Gastroenterology in a secondary-care public hospital in Durango City. Furthermore, we investigated socio-demographic, clinical, and behavioral characteristics associated with *T. gondii *seropositivity in these patients.

## Methods

### Study design and study populations

Through a case-control study design, we studied the association of liver disease with infection with *T. gondii *in adult patients and control subjects in Durango City, Mexico from January 2009 to December 2010.

#### Liver disease patients

Seventy five outpatients attended in the Gastroenterology Department of a public secondary-care hospital (Mexican Social Security Institute) in Durango City, Mexico were enrolled in the study. Forty seven patients were male and twenty eight were female. The mean age of the patients was 58.65 ± 14.41 years (range: 22-85 years). All patients resided in Durango State. Patients suffered from liver cirrhosis (n = 67), steatosis (n = 4), chronic hepatitis (n = 2), acute hepatitis (n = 1), and amoebic liver abscess (n = 1). The etiology of liver cirrhosis was alcohol consumption in 35 patients, hepatitis C virus in 4 patients, and unknown in 28 patients.

#### Control subjects

One hundred and fifty control subjects matched with patients by age, gender, and residence were included in the study. The mean age in controls was 58.68 ± 14.35 (range: 22-86) and comparable with that in patients (*P *= 0.99). Control subjects were obtained from the general population of Durango City, Mexico.

### Ethical aspects

This study was approved by the Institutional Ethical Committee of the Mexican Social Security Institute. The purpose and procedures of the study were explained to all participants, and a written informed consent was obtained from all of them.

### Socio-demographic, clinical and behavioral data

We explored socio-demographic, clinical and behavioral characteristics of the participants with the aid of a standardized questionnaire. Socio-demographic data including age, gender, birthplace, residence area, educational level, occupation, and socio-economic level were obtained from all participants. Clinical data explored in patients included type and duration of liver disease, clinical response to treatment, presence of concomitant diseases, presence or history of lymphadenopathy, frequent headache, impairments in memory, reflexes, hearing and vision, blood transfusion, transplant or surgery history. Behavioral data included animal contacts, contact with cat feces, foreign travel, kind of meat consumption (pork, beef, goat, sheep, boar, chicken, turkey, pigeon, rabbit, deer, squirrel, horse, opossum, or other), consumption of raw or undercooked meat, unpasteurized milk, dried or cured meat (chorizo, ham, sausages or salami), consumption of unwashed raw vegetables, fruits, or untreated water, frequency of eating away from home (in restaurants or fast food outlets), contact with soil (gardening or agriculture), and types of floors at home.

### Laboratory tests

Serum samples of participants were obtained and kept frozen at -20°C until analyzed. Sera were analyzed by qualitative and quantitative methods for anti-*T. gondii *IgG antibodies with the commercially available enzyme immunoassay kit "*Toxoplasma *IgG" (International Immuno-Diagnostics, Foster City, California). Anti-*T. gondii *IgG antibody levels were expressed as International Units (IU)/ml, and a result equal or greater than 8 IU/ml was considered positive. In addition, sera positive for anti-*T. gondii *IgG antibodies were further analyzed for anti-*T. gondii *IgM antibodies by the commercially available enzyme immunoassay "*Toxoplasma *IgM" kit (International Immuno-Diagnostics). All tests were performed following the instructions of the manufacturer.

### Statistical Analysis

Results were analyzed with the aid of Epi Info version 3.5.1 and SPSS 15.0 (SPSS Inc. Chicago, Illinois). Age among the groups was compared by the student's *t *test. For comparison of the frequencies among groups, the Yates corrected or, when indicated, the Fisher exact test, were used. Bivariate and multivariate analyses were used to assess the association between subject's characteristics and *T. gondii *infection. Variables were included in the multivariate analysis if they had a *P *value equal or less than 0.25 in the bivariate analysis. Odd ratio (OR) and 95% confidence interval (CI) were calculated by multivariate analysis using multiple, unconditional, logistic regression. When a cell in the 2 × 2 contingency table had a value of zero, the odds ratio was calculated by adding 0.5 to all table cells [16]. A *P *value less than 0.05 was considered statistically significant.

## Results

Anti-*T. gondii *IgG antibodies were found in 10 (13.3%) of 75 patients and in 16 (10.7%) of 150 controls (*P *= 0.71). Of the 10 anti-*T. gondii *IgG positive patients, 6 (8.0%) had IgG levels higher than 150 IU/ml, and 4 (5.3%) between 8 to 99 IU/ml. In comparison, of the 16 anti-*T. gondii *IgG positive controls, 9 (6.0%) had IgG levels higher than 150 IU/ml, 2 (1.3%) between 100 to 150 IU/ml, and 5 (3.3%) between 8 to 99 IU/ml. Anti-*T. gondii *IgG levels were comparable among patients and controls (*P *= 0.60). Anti-*T. gondii *IgM antibodies were found in 2 patients and in 5 controls (2.7% vs 3.3%, respectively; *P *= 0.57). The socio-demographic characteristics among seropositive and seronegative patients were not significantly different (Table 1). Seropositivity to *T. gondii *was significantly higher in patients with an occupation of truck driver than those with other occupations (3/3: 100% vs 8/65: 12.3%; *P *= 0.003). The type, duration and clinical response to treatment of liver disease did not show any association with the seroprevalence and levels of anti-*T. gondii *IgG (Table 2). The frequency of *T. gondii *seropositivity was higher in patients with reflex impairment (27.8%) than patients without this impairment (8.8%) (*P *= 0.05). Patients with a history of abdominal hernia repair had a significantly higher seroprevalence of *T. gondii *infection than those without this history (3/5: 60% vs 7/70: 10%, respectively; *P *= 0.01). In contrast, no statistically significant differences were observed among *T. gondii *positive and *T. gondii *negative patients in the frequencies of other clinical characteristics including concomitant diseases, frequent headaches, presence or history of lymphadenopathy, blood transfusion, or transplant, and impairments in memory, hearing or vision (Table 3).

**Table 1:** Socio-demographic characteristics of the patients and seropositivity to *T. gondii*.

			Prevalence of *T. gondii *infection		
				
Characteristic	No.	*%*	No.	*%*	*P *value
Gender					
Male	47	62.7	8	17.0	0.19
Female	28	37.3	2	7.1	
Age groups (years)					
30 or less	3	4.0	0	0.0	
31-50	17	22.7	2	11.8	0.56
51-70	41	54.7	6	14.6	
>70	14	18.7	2	14.3	
Residence place					
Durango City	75	100.0	10	13.3	
Birth place					
Durango State	67	89.3	9	13.4	0.71
Other Mexican State	8	10.7	1	12.5	
Residence area					
Urban	49	65.3	5	10.2	0.2
Suburban	1	1.3	0	0.0	
Rural	25	33.3	5	20.0	
Socio-economic level					
Low	50	72.5	8	16.0	0.44
Medium	19	27.5	2	10.5	
Educational level					
No education	6	8.0	1	16.7	0.58
Up to 6 years	63	84.0	8	12.7	
7-12 years	6	8.0	1	16.7	
Occupation					
No laborer^b^	26	34.7	1	3.8	0.07
Laborer^c^	49	65.3	9	18.4	

^b^Non laborer = none occupation, student or housewife.

^c^Laborer = Employee, business, agriculture, construction worker, driver or other.

**Table 2:** Bivariate analysis of liver disease characteristics in patients and seropositivity to *T. gondii *infection.

	No. of subjects tested	Prevalence of *T. gondii *infection		*P *value	Anti-*T. gondii *IgG levels >150 IU/ml	
				
Characteristic		No.	%		No.	%
Diagnosis						
Acute hepatitis	1	0	0.0		-	
Chronic hepatitis	2	0	0.0		-	
Cirrhosis	67	9	13.4	0.89	5	55.6
Steatosis	4	1	25.0		1	100
Amoebic abscess	1	0	0.0		-	
Alcohol related disease						
Yes	35	6	17.1	0.28	3	50
No	40	4	10.0		3	75
Duration of disease						
Less than 1 year	30	5	16.7	0.35	3	60
1 to 5 years	34	4	11.8		3	75
More than 5 years	11	1	9.1		1	100
Treatment response						
Good	50	8	16.0		4	50
Regular	4	1	25.0		1	100
Bad	3	1	33.3	0.44	1	100

**Table 3:** Bivariate analysis of clinical data and infection with *T. gondii *in patients.

	No. of subjects tested	Prevalence of *T. gondii *infection		*P *value
Characteristic		No.	%	
Concomitant disease				
Yes	49	5	10.2	0.22
No	26	5	19.2	
Lymphadenopathy ever				
Yes	11	1	9.1	0.54
No	64	9	14.1	
Headache frequently				
Yes	25	3	12	0.55
No	50	7	14	
Blood transfusión				
Yes	50	6	12	0.44
No	25	4	16	
Transplantation				
Yes	4	0	0	0.55
No	71	10	14.1	
Surgery ever				
Yes	42	7	16.7	0.27
No	33	3	9.1	
Memory impairment				
Yes	32	4	12.5	0.56
No	43	6	14	
Reflex impairment				
Yes	18	5	27.8	0.05
No	57	5	8.8	
Hearing impairment				
Yes	48	6	12.5	0.51
No	27	4	14.8	
Visual impairment				
Yes	27	3	11.1	0.48
No	48	7	14.6	

Bivariate analysis showed a number of behavioral characteristics with a *P *value equal or less than 0.25 including cats at home, raising animals, traveling abroad, consumption of sheep, chicken, turkey, pigeon, rabbit, deer, squirrel, quail, skunk and armadillo meats, consumption of raw milk, ham, unwashed raw fruits, and untreated water, soil contact and soil floors at home. Multivariate analysis of these behavioral characteristics showed that consumption of sheep meat (OR = 8.69; 95% CI: 1.02-73.71; *P *= 0.04), rabbit meat (OR = 4.61; 95% CI: 1.06-19.98; *P *= 0.04), venison (OR = 40.46; 95% CI: 2.25-725.75; *P *< 0.01), and quail meat (OR = 38.50; 95% CI: 1.70-871.99; *P *< 0.01) were significantly associated with *T. gondii *infection in patients (Table 4). Other behavioral characteristics did not show an association with *T. gondii *infection. Raw data of patients and controls are included in additional files [additional file 1-cases and additional file 2-controls, respectively].

**Table 4:** Multivariate analysis of selected characteristics of patients and their association with *T. gondii *infection.

	Raw numbers				
				
Characteristic	Yes	No	Odds ratio	95% Confidence interval	*P *value
Cats at home	7/41	3/34	1.99	0.45 - 8.81	0.36
Raising animals	8/48	2/27	2.53	0.48 - 13.37	0.27
Traveling abroad	6/34	4/41	2.24	0.54 - 9.23	0.26
Sheep meat consumption	9/43	1/32	8.69	1.02 - 73.71	0.04
Chicken meat consumption^a^	9/74	1/1	0.04	0.001-1.27	0.01
Turkey meat consumption	9/51	1/24	5.97	0.68 - 51.93	0.10
Pigeon meat consumption	3/12	7/63	2.42	0.46 - 12.69	0.29
Rabbit meat consumption	7/31	3/44	4.61	1.06 - 19.98	0.04
Venison consumption^a^	10/36	0/39	40.46	2.25-725.75	<0.01
Squirrel meat consumption	5/24	5/51	2.46	0.59 - 10.29	0.21
Quail meat consumption^a^	2/2	8/73	38.50	1.70-871.99	<0.01
Skunk meat consumption	2/5	8/70	3.11	0.43 - 22.39	0.25
Armadillo meat consumption	1/2	9/73	7.11	0.08-566.52	0.05
Raw milk consumption	6/31	4/44	2.26	0.56 - 8.99	0.24
Ham consumption	7/63	3/12	0.46	0.09 - 2.21	0.33
Unwashed raw fruits	5/24	5/51	2.4	0.60 - 9.58	0.21
Untreated water	9/47	1/28	6.73	0.75 - 60.49	0.08
Soil contact	9/53	1/22	3.86	0.45 - 33.17	0.21
Soil floor at home	4/16	6/59	2.35	0.51 - 10.72	0.26

^a^Odd ratios for these characteristics were calculated by adding 0.5 to each cell of the 2 × 2 table.

## Discussion

In this seroprevalence case-control study, we found a comparable frequency of anti-*T. gondii *IgG and IgM antibodies in liver disease patients and controls. Similarly, levels of anti-*T. gondii *IgG antibodies were comparable among these groups indicating that *T. gondii *infection is not likely to substantially contribute to the etiology of liver disease in our patient population. We are not aware of previous reports about the association of *T. gondii *infection in liver disease patients in Mexico, and reports in other countries are scarce. Our results conflict with those reported in a Turkish study where researchers found an association of *T. gondii *infection with liver cirrhosis [15]. Most of our patients suffered from liver cirrhosis but we did not find any association between seropositivity to *T. gondii *and cirrhosis. Similarly, the comparable seroprevalence of *T. gondii *infection in patients and controls differs from those reported in an Egyptian study where researchers found a 65.5% seroprevalence of *T. gondii *antibodies in patients with acute and chronic hepatic diseases against a 27% seroprevalence found in controls [17]. Certainly, differences in the characteristics of the studies might explain the differences in the seroprevalences including the use of different laboratory methods and matching procedures, difference in ages of participants and proportions of controls and patients.

None of the socio-demographic characteristics and diagnosis of liver disease associated with *T. gondii *seropositivity in our patients. Concerning behavioral characteristics, it was noteworthy that there was an association between *T. gondii *seropositivity and sheep meat consumption (OR = 8.69; 95% CI: 1.02-73.71; *P *= 0.04). Infections with *T. gondii *have been reported in sheep [18]. In addition, viable *T. gondii *has been found in lambs destined for meat consumption in the USA [19], and ovine meat consumed in France [20]. It will therefore be of interest to examine the seroprevalence of *T. gondii *infection in sheep in Durango. In a recent study in the USA, elevated risk of recent *T. gondii *infection was associated with eating rare lamb [21]. Even frozen lamb meat has been associated with acute *T. gondii *infection in Brazil [22]. The association of *T. gondii *infection and consumption of sheep meat in our patients was unexpected since lamb meat consumption was negatively associated with *T. gondii *infection in a previous study in psychiatric patients in Durango [23]. We are not aware of any previous report about a positive association of *T. gondii *infection and consumption of sheep meat in Mexico. Remarkably, consumption of rabbit meat was also associated with *T. gondii *infection in patients (OR = 4.61; 95% CI: 1.06-19.98; *P *= 0.04). Infections with *T. gondii *in rabbits have been reported in several countries [24-26]. Antibodies against *T. gondii *were found in 77 (26.9%) of 286 domestic rabbits from three rabbit farms in Mexico [25]. However, the seroprevalence of *T. gondii *infection in rabbits in Durango is unknown. To the best of our knowledge there is not any previous report about the association of *T. gondii *infection and consumption of rabbit meat. In the present study, we also found an association between *T. gondii *seropositivity and consumption of quail meat (*P *< 0.01). Experimental infections with *T. gondii *in bobwhite [27] and Japanese quail [28] have been reported. We are not aware of any previous epidemiological report about the association of *T. gondii *infection and consumption of quail meat. More expectedly, we observed an association between *T. gondii *seropositivity and consumption of venison (*P *= < 0.01). Infections with *T. gondii *have been reported in deer [29,30]. Consumption of undercooked or uncooked venison has been linked to ocular toxoplasmosis in deer hunters [31]. Interestingly, toxoplasmosis with liver involvement has been reported in deer hunters who had eaten undercooked venison [32].

The frequency of *T. gondii *seropositivity was higher in patients with reflex impairment (27.8%) than patients without this impairment (8.8%), and this difference showed a borderline significance (*P *= 0.05). In a previous study in patients with visual impairment in Durango, we found that patients with reflex impairment had a significantly higher frequency of *T. gondii *infection than those with normal reflexes [33]. Reflex impairment might contribute to reducing the quality of life in *T. gondii *infected patients.

## Conclusions

Seropositivity to *T. gondii *was comparable among liver disease patients and controls. Further studies with larger sample sizes are needed to elucidate the association of *T. gondii *with liver disease. Consumption of venison, and rabbit, sheep, and quail meats may warrant further investigation.

## Competing interests

The authors declare that they have no competing interests.

## Authors' contributions

CAE conceived and designed the study protocol, participated in the coordination and management of the study, applied the questionnaires, performed the laboratory tests and data analysis, and wrote the manuscript. JLTB and MFMS obtained clinical data, applied the questionnaires and performed the data analysis. SEM performed the statistical analysis. OL performed the data analysis, and wrote the manuscript. All authors read and approved the final manuscript.

## Funding

This study was supported by Fondos Mixtos Durango-Consejo Nacional de Ciencia y Tecnología, Mexico. Grant No. 66718.

## Supplementary Material

Additional file 1 **Patients**. Raw data of patients suffering from liver diseases. Click here for file

Additional file 2 **Controls**. Raw data of controls of patients suffering from liver diseases. Click here for file
